# Vehiculation of Active Principles as a Way to Create Smart and Biofunctional Textiles

**DOI:** 10.3390/ma11112152

**Published:** 2018-11-01

**Authors:** Manuel J. Lis Arias, Luisa Coderch, Meritxell Martí, Cristina Alonso, Oscar García Carmona, Carlos García Carmona, Fabricio Maesta

**Affiliations:** 1 Textile Research Institute of Terrassa (INTEXTER-UPC), 08222 Terrassa, Spain; oscargarciacarmona@gmail.com (O.G.C.); carlos.garcia.carmona@gmail.com (C.G.C.); 2 Catalonia Advanced Chemistry Institute (IQAC-CSIC), 08034 Barcelona, Spain; luisa.coderch@iqac.csic.es (L.C.); mmgesl@iiqab.csic.es (M.M.); cristina.alonso@iqac.csic.es (C.A.); 3 Textile Engineering Dept., Federal Technological University of Paraná, Apucarana 86812-460, Brazil; fabriciom@utfpr.edu.br

**Keywords:** microencapsulation, biofunctional, drug-delivery

## Abstract

In some specific fields of application (e.g., cosmetics, pharmacy), textile substrates need to incorporate sensible molecules (active principles) that can be affected if they are sprayed freely on the surface of fabrics. The effect is not controlled and sometimes this application is consequently neglected. Microencapsulation and functionalization using biocompatible vehicles and polymers has recently been demonstrated as an interesting way to avoid these problems. The use of defined structures (polymers) that protect the active principle allows controlled drug delivery and regulation of the dosing in every specific case. Many authors have studied the use of three different methodologies to incorporate active principles into textile substrates, and assessed their quantitative behavior. Citronella oil, as a natural insect repellent, has been vehicularized with two different protective substances; cyclodextrine (CD), which forms complexes with it, and microcapsules of gelatin-arabic gum. The retention capability of the complexes and microcapsules has been assessed using an in vitro experiment. Structural characteristics have been evaluated using thermogravimetric methods and microscopy. The results show very interesting long-term capability of dosing and promising applications for home use and on clothes in environmental conditions with the need to fight against insects. Ethyl hexyl methoxycinnamate (EHMC) and gallic acid (GA) have both been vehicularized using two liposomic-based structures: Internal wool lipids (IWL) and phosphatidylcholine (PC). They were applied on polyamide and cotton substrates and the delivery assessed. The amount of active principle in the different layers of skin was determined in vitro using a Franz-cell diffusion chamber. The results show many new possibilities for application in skin therapeutics. Biofunctional devices with controlled functionality can be built using textile substrates and vehicles. As has been demonstrated, their behavior can be assessed using in vitro methods that make extrapolation to their final applications possible.

## 1. Introduction

In November 2017, the title of the International Symposium on Materials from Renewables (ISMR) was “Advanced, Smart, and Sustainable Polymers, Fibers and Textiles”. Three specific sessions occurred under the denomination of “Smart Fibers and Textiles”. That simple fact gives an idea of the importance of this work. However, what really are smart textiles? In the foreword of the book edited by Tao, X. [1], Lewis states clearly that these type of textiles are not only special finished fabrics. The main defining idea of smart textiles is related to the “active character” of them. Smart textiles “react to environmental *stimuli*, from mechanical, thermal, chemical, magnetic or others”, including biotechnology, information technology, microelectronics, wearable computers, nanotechnology, and micromechanical machines.

Biofunctional textiles are fibrous substrates that have been modified to attain new properties and added value. The main idea is to modify their parameters, especially related to comfort, adapting the tissues’ reaction to external or internal stimuli. Such textiles constitute appropriate substrates to be used for the delivery of active principles in cosmetic or pharmaceutical applications. Due to their specific response, biofunctional textiles are especially useful when the textile comes into close contact with the skin. As most of the human body is covered with some sort of textile, the potential of this type of textile is considerable. Textiles with functional properties used for delivery to skin have been studied and patented [2,3].

Three cases will be explored in this work as examples of biofunctional systems obtained using vehicles to transport different active principles to a textile substrate: Microcapsules, cyclodextrins, and liposomes.

### 1.1. Microcapsules

Microcapsules may be obtained by a series of techniques that involve liquids, gases, or solids in natural or synthetic polymeric membranes [4,5,6,7]. This process is known as microencapsulation, and requires a layer of an encapsulating agent, generally a polymeric material, that acts as a protective film insulating the active substance [8,9,10].

According to Souza and collaborators [11], this creates a physical barrier between the core (active principle) and the encapsulating material (shell).

This membrane is removed by a specific stimulus, releasing the active substance in the ideal place or moment.

The encapsulation technique can be used to fulfill diverse objectives, and has the following advantages, as listed by different authors [12,13,14]: Protection of the encapsulated materials against oxidation or deactivation due to reaction with the environment (light, oxygen, humidity); masking odors, tastes, and other active principles; insulation of the active principles of undesirable materials; retardation of alterations that might occur in loss of aroma, color, and flavor; separation of reactive or incompatible components; and reduction of the migration rate of the core to the external environment.

For this reason, the core and shell should have compatible physical-chemical properties [15].

The materials commonly used as encapsulating agents (shells) are polymers [16,17] and biologic-based materials [18,19,20].

Bosnea and collaborators [21] highlighted that shell materials based on natural polymers are promising for the formation of microcapsules due to their biodegradability, compatibility with other products, and low toxicity, as well as their wide availability from natural resources.

Furthermore, Al Shannaq and collaborators [22] accentuate in their work that the choice of encapsulation material is important to obtain better degradation of the microcapsule. Table 1 shows the main polymers of natural sources used in microencapsulation.

Besides the inherent parameters of the shell material choice, Santos, Ferreira, and Grosso [28] pointed out that the retention of the active principle is a fundamental factor for the realization of the process. Wang et al. [29] and Yang et al. [30] demonstrated in their works that the efficiency of the encapsulation should be a parameter that is taken into account in the choice of the core.

Sharipova et al. [31] showed that the value of the effectiveness of the encapsulation depends on the encapsulating method, the shell material, and the relationship with the core. Thus, the casing material should relate to the chemical nature (molar mass, polarity, functionality, volatility, and so forth) of the active principle so that a high yield of the process is possible.

There are several techniques for obtaining microcapsules, which can be divided into physical methods (spray drying [32], solvent evaporation [33], pan coating [34]); chemical methods (interfacial polymerization [35], suspension polymerization [36], in situ polymerization [37]); and physical-chemical methods (coacervation [38], ionic gelation [39], sol-gel [40]), among others.

The microencapsulation technique selection depends on the properties of the active principle, the morphology of the desired particle, the nature and capacity of releasing the components, reproducibility, ease of execution of the technique, and the cost/effectiveness ratio [41]. The chosen technique is a determining factor of the characteristics of the formed microcapsule, and will influence the release of the encapsulated agent via one of the following actions: Mechanical, temperature, pH, dissolution, or biodegradation [42].

Microcapsules in the textile field have been applied in various ways, giving very interesting results and showing very promising applications in several fields, including the use of flame retardant agents [43], protection of atmospheric agents [44,45,46], and functional finishing [47,48,49,50], along with the development of functional fabrics that might have a useful effect on the user and solve problems that conventional processes are not able to [51,52].

Nowadays, microcapsules are applied in textiles to transmit different embedded values, such as the liberation of oils with medicinal effects, protection against disease carriers, and antimicrobials, among others [53,54,55,56,57,58,59,60,61,62,63,64,65].

Microencapsulation of citronella oil, to be used as insect repellent and obtained by the coacervation method with the gelatin-arabic gum system, will be detailed as an example [38].

To assess the results obtained by the methodology applied, instrumental techniques will be used. Thermogravimetric analysis will be undertaken (TGA/DTGA), as well as application to textile fabrics by padding and analysis of the delivery kinetics. The results will show their potential for use as vehicles.

### 1.2. Cyclodextrines (CDs)

Cyclodextrines (CDs) are used in diverse industrial fields, such as food, drugs, cosmetics, domestic products, agrochemicals, the textile industry, the paper industry, chemical technology, and others [66,67,68]. This wide range of applications can be attributed to the fact that cyclodextrins have the capacity to form inclusion complexes with a broad range of substances, allowing the alteration of important properties in the complexed substances [69].

According to Matioli and collaborators [70], CDs are regularly produced from starch by the cyclation of linear chains of glucopyranoses using the enzyme cyclomaltodextrin-glucanotransferase (CGTase).

The three widest known natural cyclodextrins are alpha CD (α-CD), beta CD (β-CD), and gamma CD (γ-CD), composed of six, seven, and eight units of d-(+)-glucopyranose, respectively, and united by α-1,4 bonds.

Since the first publication regarding cyclodextrins in 1891, and the first patent in 1953, these molecules have been a source of great interest to researchers [71]. Nowadays, the widest use of cyclodextrins is their complexation with many classes of drugs [72,73,74,75,76], flavors [77,78,79], and aromas [80,81,82].

In order to compare them with the microencapsulation system, complexes of CDs will also be used to vehicularize citronella oil. The results will help us to understand how both systems may show possibilities for use on textile substrates to deliver this active compound, to be used as an insect repellent vector.

### 1.3. Liposomes

Liposomes are vesicles prepared with lipids that can encapsulate different ingredients; one of their applications could be onto textiles. Due to the liposome bilayer structure, liposomes have been applied as models for biological membranes in medical research. Another important use of this type of vesicle is as microcapsules for drug delivery in the cosmetic field [83,84,85,86]. The textile industry has used liposomes in the wool dyeing process as an auxiliary [87,88]. Liposomes could have different properties depending on the lipidic base used during their formulation. In this study, phosphatidylcholine (PC) and internal wool lipids (IWL) were used.

Internal wool lipids are a mix of cholesterol, free fatty acids, cholesterol sulphate, and ceramides, similar to those found in membranes of other keratinized tissues such as human hair or stratum corneum (SC) from skin. Wool is a fiber which is mainly keratinic but with a small amount of internal lipids [89,90]. Due to the IWL liposome bilayer structure’s similarity to stratum corneum lipids, their application onto human skin has been assessed in previous studies. The results obtained have demonstrated the beneficial effect of this type of liposome when used with ceramides, topically applied onto intact skin in aging populations or in individuals with dry skin [91,92,93]. Therefore, we could consider IWL liposomes an optimal encapsulation route for cosmetic or dermopharmacy applications [94].

Using a solar filter as a tracer, ethyl hexyl methoxycinnamate (EHMC), PC-based, and IWL-based liposomes were prepared. The influence of the type of lipid in the vesicle on skin penetration has been demonstrated in previous studies. In particular, the crystalline liquid state of PC liposomes seems to play an important role in this characteristic. On the other hand, when using IWL liposomes, penetration into the skin is delayed—a fact that suggests some reinforcement of the barrier function of the skin’s stratum corneum [95]. These two types of liposomes, with IWL and PC, were chosen to be applied to cotton and polyamide fabrics to design biofunctional textiles.

To evaluate the effectiveness of the textile in contact with the skin, a series of methodologies and an in vitro process were optimized to determine the penetration of the encapsulated active principle.

For the evaluation of the biofunctional textiles’ beneficial capacity on skin, the transepidermal water loss change (TEWL) was used as an indicator of the barrier function state. TEWL measures water-holding capacity as changes in skin capacitance [95]. An in vitro methodology based on percutaneous absorption [96] was used to determine the amount of encapsulated principle that passed into the different skin layers (stratum corneum, epidermis, or dermis) from the textile.

An in vivo stripping was used as a minimally invasive methodology, where a series of strippings allowed quantification of the amount of active principal in the outermost layers of the SC [97].

These methodologies have shown that liposomes, especially IWL liposomes, are suitable for applying active principles onto biofunctional textiles.

## 2. Materials and Methods

### 2.1. Microcapsules

#### 2.1.1. Materials

The materials used to produce the microcapsules were type A gelatin (GE, Sigma Chemical, Darmstadt, Germany) and arabic gum (GA, Sigma Chemical, Darmstadt, Germany), which were used as the microcapsule walls. Citronella essential oil (WNFt, Sao Jose dos Campos, Brazil) was used as the core material. Glutaraldehyde (50%), sodium lauryl sulfate (SLS), citric acid, sodium hydroxide, and all other chemicals used were of analytical grade. The textile substrate consisted of standard cotton fabric (COT, bleached and desized, style 400, 100 g cm^−2^) and spun polyester type 54 fabric (PES, Style 777, 126 g m^−2^). Both were obtained from Test Fabrics Inc. (West Pittston, PA, USA).

Optical microscopy was used to verify shape and homogeneity. A BX43 (Olympus, Tokyo, Japan) equipment and a JEOL-JSM 5610 (JEOL, Ltd., Tokyo, Japan) scanning electron microscope were used to analyze the distribution of microcapsules on the textile. The thermal characteristics of the microcapsules were studied by TGA (TGA, SDTA851, Mettler Toledo, Columbus, OH, USA) with the STARe software (version SW 9.01, Mettler-Toledo, Columbus, OH, USA). The samples were weighed and examined in the temperature range of 30–800 °C, using a heating rate of 10 °C min^−1^.

#### 2.1.2. Methodology

The procedure began with the formation of three emulsions. The first contained 3 g gelatin in 50 mL water, the second emulsion contained 5 mL citronella essential oil and 0.3 g sodium lauryl sulfate (SLS), and the last emulsion was prepared with 100 mL of water and 3 g of gum arabic. The three emulsions were prepared separately in aqueous solution at 50 °C and under stirring at 300 rpm, and the ratio GA:GE was 1:1, as has been used in several other works [38].

In the next stage, the first and second emulsions were blended. Then, the third emulsion was added to the previous mixture followed by adjustment of pH to 4 using citric acid. The mixture was left at rest for 90 min until complete stabilization. The resulting suspension was cooled to a temperature of <8 °C and remained unperturbed for 1 h. Then, the pH of this preparation was adjusted to 8–9 using NaOH (1 M), and 1 g glutaraldehyde (50%) was added dropwise. The system was left for 12 h under stirring at room temperature, resulting in microcapsules for application into fabric substrates.

### 2.2. Cyclodextrin Complexes

#### 2.2.1. Materials

β-cyclodextrin (CD) was supplied by Wacker-Chemie GmbH, Germany, and citronella essential oil by WNFt, Brazil. Butane 1,2,3,4 tetracarboxylic acid (BTCA) and sodium hypophosphite monohydrate (SHP) were both supplied by Sigma-Aldrich, São Paulo, Brazil, and were of analytical grade.

Standard woven fabrics, namely cotton fabric (COT, bleached and desized cotton print cloth, style 400, 100 g m^−2^, ISO 105-F02) and spun polyester type 54 fabric (PES, style 777, 126 g m^−2^, ISO 105-F04) were used, both supplied by Test Fabrics Inc. (West Pittston, PA, USA).

#### 2.2.2. Methodology

A solution of 50 mL of ethanol and water (1:3) and 3 g of CD was prepared and emulsified at 18,000 rpm for 5 min at 60 °C. High temperature and strong agitation allows cyclodextrin to become entirely soluble. Subsequently, 5 g of citronella oil was added. After 2 h agitation, the complexes are formed. This is a standard process proposed by several authors, including Wang and Cheng [61], Oliveira et al. [98], and Partanen et al. [81].

Application of the complexes to the fabrics was achieved by the pad-dry process. Dehabadi, Buschman and Gutmann [99] proposed following the application with a curing step at 170 °C for 3 min, to complete the process of fixation of the complexes by cross-linking.

The pad-dry machine had a width of 30 cm (ERNST BENZ AG KLD-HT and KTF/m250), and a pick-up of 90 ± 10%. The textile substrates were left to dry in environmental conditions.

COT and PES textile articles were impregnated for 1 min in 6 g L^−1^ cyclodextrin solution. The crosslinking agent used was butane 1,2,3,4 tetracarboxylic acid (BTCA) 6 g L^−1^ and the catalyst used was sodium hypophosphite at 6 g L^−1^.

### 2.3. Liposomes

#### 2.3.1. Materials

The standard fabrics used were plain cotton fabric, (bleached and desized cotton print cloth, style 400 ISO 105-F02), spun nylon 6 Du Pont type 200 woven fabric (PA) (style 361, 124 g/m^2^,) spun polyester type 54 fabric (PES) (style 777, 126 g/m^2^) (2,3,8), spun polyacrylic fabric (PAC) (style 864, 135 g/m^2^), and knitted 100% wool fabric (WO) (style 537). The textile bandages used for skin penetration evaluation were knitted fabrics (plain stitch) of polyamide 78/68/1 (DeFiber, S.A., Tarragona, Spain).

Ethylhexhyl methoxycinnamate (EHMC) (Escalol 557, ISP Global Headquarters, Wayne, NJ, USA) was used as the active principle, as a sun filter and as a tracer in the liposome preparation.

An antioxidant active principle, gallic acid (GA) (Sigma-Aldrich, São Paulo, Brazil), was employed, and isopropanol (Carlo Erba, Cornaredo, Italy) was used in its detection.

#### 2.3.2. Methodology

The liposomes were prepared using two kinds of lipids: Internal wool lipids (IWL) and phosphatidylcholine (PC). The internal wool lipids are a mixture of cholesterol-esters (4%), free fatty acids (27%), cholesterol (19%), glycosylceramides (12%), cholesteryl sulfate (10%), and ceramides (29%). For this study, they were obtained in an extraction pilot plant [93,100].

The method used to prepare liposomes was film hydration, in which the first step was lipid solubilization in an organic phase. Secondly, the lipid solution was dried under a vacuum to form a lipid film on the flask wall, and third, a dispersed water solution of the active principle was added and with movement (energy) the vesicles were formed.

Liposomes of 4% Emulmetik 900 (PC) and 2% of the active principle (gallic acid (GA) or sun filter (EHMC)), were prepared using the well-known method of film hydration. PC (4 g) solubilized in 30 mL of chloroform was dried under a vacuum. The lipidic film was dispersed in 100 mL of 2% GA aqueous solution and then multilamellar vesicles (MLV) were obtained.

Liposomes prepared with phosphatydilcholine or internal wool lipids were also prepared to apply onto textiles. 

In order to quantify the exact amount of GA encapsulated, 1 mL of liposome formulation was precipitated and separated from the supernatant by centrifugation using a 5415-Eppendorf centrifuge at 14,000 rpm for 15 min. After separation, the supernatant was retained. The initial and the supernatant were diluted in methanol and read spectrophotometrically at 269 nm. The entrapment efficacy of GA in the microspheres was determined by the amount of the active ingredient present in the whole solution, as well as in the supernatant. 

Liposomes with active principle were applied by the foulard process using a pad-dry machine with a measured width of 30 cm (ERNST BENZ AG KLD-HT and KTF/m250), with the corresponding rolling pressure to obtain a pick-up of 90 ± 10% ((mass of bath solution taken by the textile/mass of dry textile) × 100). This was followed by drying in a curing and heat-setting chamber.

#### 2.3.3. Assessment of the Absorption of Skin

Due to the specific skin-delivery applications of the vehicles formed using liposomes, the assessment of the final product’s properties was different from the techniques used in former cases. The technique used in this case was percutaneous absorption, using a Franz Diffusion cell, which can be considered as an in vitro assessment in the field of skin care cosmetics.

Pig skin was used from the unboiled backs of large white/Landrace pigs weighing 30–40 kg. The pig skin was provided by the Clínic Hospital of Barcelona, Spain. After excision, the skin was dermatomed to a thickness of approximately 500 ± 50 μm with a Dermatome GA630 (Aesculap, Tuttlingen, Germany). Skin discs with a 2.5 cm inner diameter were prepared and fitted into static Franz-type diffusion cells.

**Figure 1:**
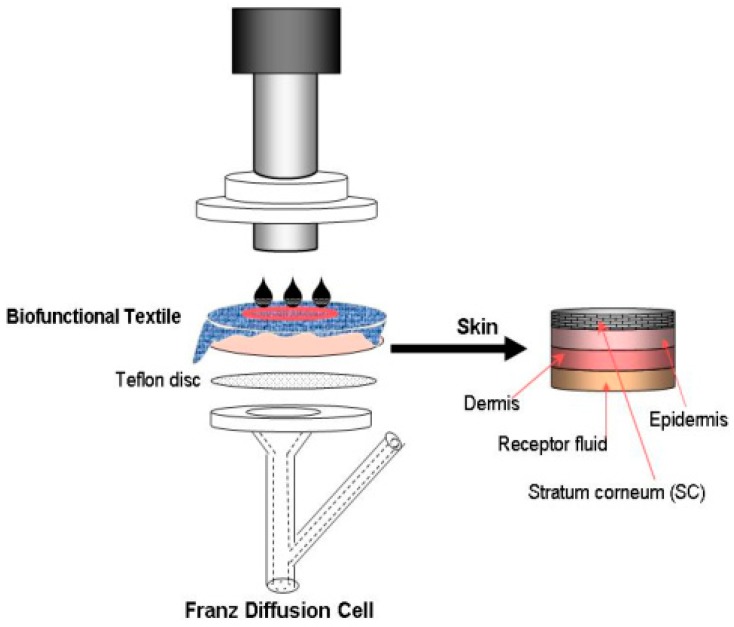
Diagram of in vitro percutaneous absorption experiments.

Skin absorption studies were initiated by applying 10 µL of GA in liposomes, or by applying the textile substrates treated with the same formulations onto the skin surface. Both types of samples were allocated in the Franz-cell system. Between the textile and the skin, 20 μL of distilled water was added to ensure close contact. According to the OECD methodology [20], the skin penetration studies were performed for 24 h of close contact between the textile and the skin. To increase the contact pressure between the textile fabric and skin, permeation experiments were also carried out by placing a steel cylinder on the textile-skin substrate at a constant pressure, in accordance with standard conditions (125 g/cm^2^) (see Figure 1).

After the exposure time, the receptor fluid was collected and brought to a volume of 5 mL in a volumetric flask. In the case of the formulations, the skin surface was washed with a specific solution (500 μL SLES (sodium lauryl ether sulphate) (0.5%), and twice with 500 μL distilled water) and dried with cotton swabs. In the case of the textiles, the fabrics were removed from the skin surface and collected together with the top of the cell. In both cases, after eliminating the excess of actives from the skin surface, the stratum corneum of the skin was removed using adhesive tape (D-Squame, Cuderm Corporation, Dallas, TX, USA) applied under controlled pressure (80 g/cm^2^ for 5 s). The epidermis was separated from the dermis after heating the skin to 80 °C for 5 s.

The actives were extracted from the different samples (surface excess, CO/PA, or skin layers) using a methanol:water (50:50) solution agitated in an ultrasonic bath for 30 min at room temperature. The receptor fluids were directly analyzed. After filtration on a Millex filter (0.22 μm, Millipore, Bedford, MA, USA), the solutions were assessed by HPLC-UV.

##### In Vivo Methodologies

In vivo methodologies were applied not only to evaluate the penetration of actives into the skin using the stripping technique, but also to determine the effectiveness of the actives, mainly using biophysical techniques.

The cosmeto-textiles (cotton or polyamide with EHMC in IWL/PC liposomes polyamide containing ME-GA, and control textiles (cotton or polyamide)) were applied on the subjects’ arms, maintaining close contact with the skin over a period of four days as a bandage application. The volar forearm was previously evaluated by non-invasive biophysical techniques: Tewameter TM300 (Courage + Khazaka, Köln, Germany) and Corneometer CM825 (Courage + Kazaka, Köln, Germany). Both parameters (TEWL and skin hydration) were recorded in accordance with established guidelines. After 24 h the textiles were removed and these skin properties were again determined.

The tape stripping of the SC of each forearm and arm was carried out on the fourth day in a conditioned room at 25 ± 1 °C and 50 ± 2% relative humidity using D-Squame tape (φ = 22 mm, CuDerm, Dallas, TX, USA) previously pressed onto the skin with a roller and stripped in one quick move. The weight of the SC removed was determined. The SC lipids from a group of three strips were extracted with methanol (Merck, Darmstadt, Germany) and sonicated in a Labsonic 1510 device (B. Braun, Melsungen, Germany) for 15 min, and the solutions were assessed by HPLC-UV.

## 3. Results and Discussion

### 3.1. Microcapsules

#### 3.1.1. Microscopy: Optical and SEM

**Figure 2:**
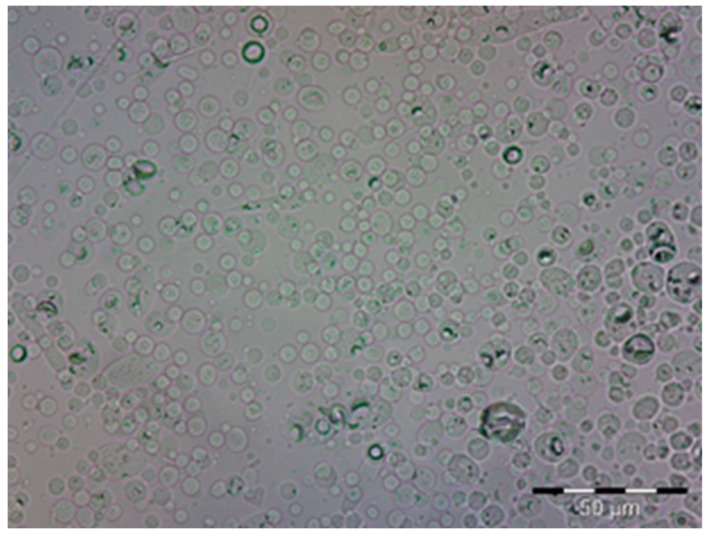
Optical microscopy image of microcapsules formed by complex coacervation (magnification 1000×).

The first approach used to detect the existence of microcapsules was microscopy. As can be seen in Figure 2, the profile of microcapsules is easy to distinguish in the picture that was obtained.

From the image in Figure 2, it is possible to detect the uniform distribution of the microcapsules. There is a clear interface between them and the environment.

#### 3.1.2. TGA and DTGA

**Figure 3:**
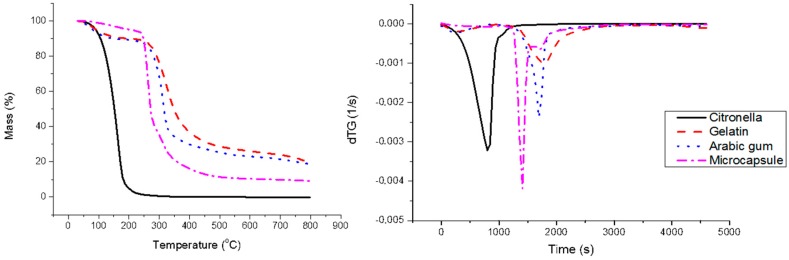
TGA/DTGA thermogram for different components of the system.

The thermogravimetric curves shown in Figure 3 reveal that each component of the system has a different loss of weight when submitted to temperature changes in the oven of the instrument. Citronella oil shows a single stage loss of weight, while gelatin and arabic gum show two stages. Formation of the microcapsule gives the new organized system that presents three different stages of thermal rupture. This behavior is equivalent to that observed by Otálora et al. [101]. From these thermograms it can be seen that the microcapsule was formed, as was already reported in other papers by some of the same authors [38].

#### 3.1.3. Application to PES and COT Fabrics Using Pad-Dry

**Figure 4:**
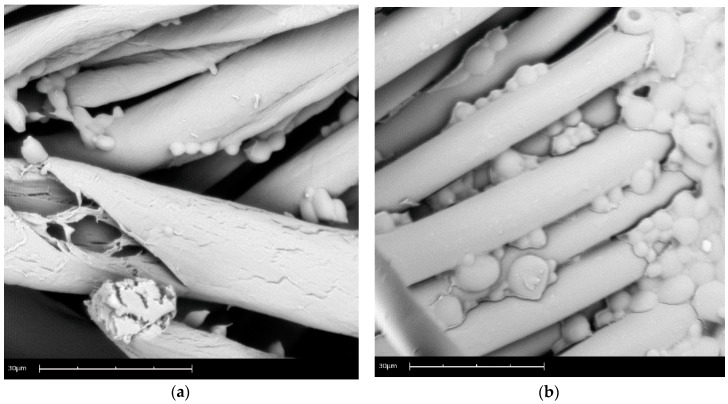
Scanning electron of microcapsules: (**a**) cotton, and (**b**) polyester.

Figure 4 shows the scanning electron microscopy of the microcapsules and applied cotton (a) and polyester (b). In these micrographs it is possible to verify the distribution of the dispersed microcapsules and their reduced size. These results were also observed in the work of Vahabzadeh, Zivdar, and Najafi [102].

Another factor that can be pointed out is the small size of the microcapsules, which facilitates the absorption and penetration of the fabric surface due to the occupation of the interstices of the textile article. Li et al. [23] related the advantages of controlled dosing and increased durability of the textile finish to this small size.

It is also evident that the microcapsules ensure effective protection of the encapsulated material, as already shown by the results of TGA. In short, it can be noted that the encapsulated material is not exposed to the elements, which has been the most serious problem of the application of oils in textiles, as pointed out by Chinta and Pooja [51] and Nelson [42].

#### 3.1.4. Drug Delivery Kinetics

To assess the complexation process yield, spectroscopy at the UV region of the citronella oil in acetone solution was undertaken in order to detect the wavelength of maximum absorption (wavelength from 250 to 550 nm).

These many possibilities of fabric modification allow the textile to be combined with the controlled release system, thereby enabling the absorption of therapeutic or cosmetic compounds and the release of them to the skin [48,103,104,105,106].

**Figure 5:**
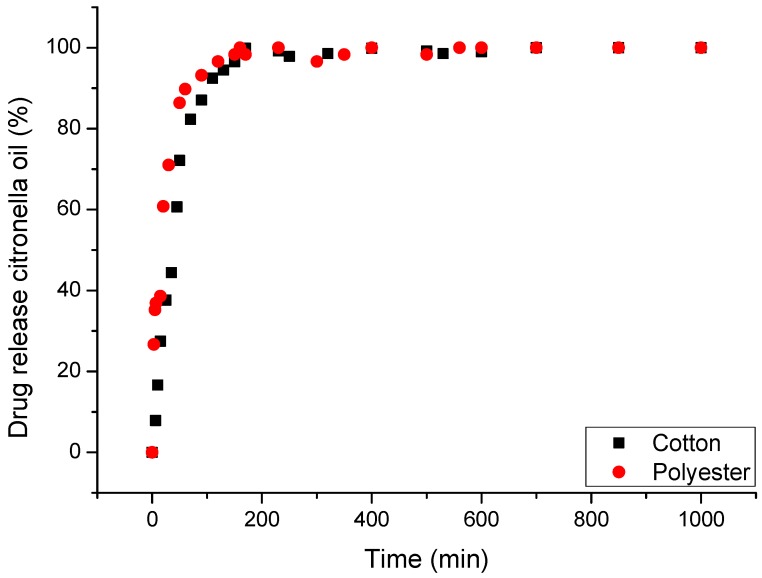
In vitro controlled release profiles in water at 37 °C for microencapsulated citronella oil applied on textiles.

The behavior observed in Figure 5, in the rate of release in water, allows us to affirm that until 200 min the retention of citronella oil is good enough to state that the delivery is controlled by the structure of the microcapsule. That means, as stated previously, that the structure established clear interactions between the microencapsulated oil (core) and the polymers used to build the microcapsule wall (shell). The nature of these interactions is related to chemical affinity. The majority of the components of the oil have a very organic character that makes them compatible with the organic part of polymers. Although these interactions are weak (see the high slope in Figure 5, compared with that obtained in the case of CD’s), the compacted structure of the shell allows the different components of the oil to be retained.

**Figure 6:**
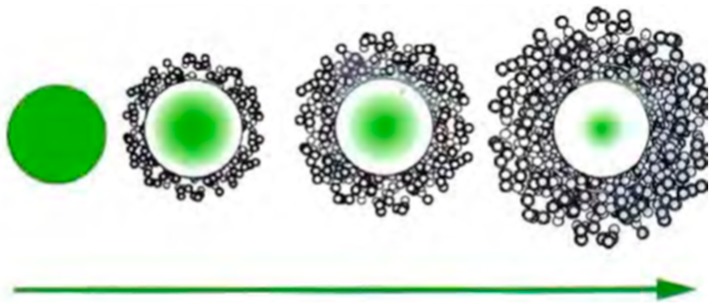
Schematic representation of the diffusion of an active system with the polymeric membrane, adapted from Bezerra [108].

In this case, the mechanism is diffusion-controlled, as shown in Figure 6. The rate of absorption of water is the determining step of the delivery to start transporting citronella oil to the bath, as was established previously in several other works [107].

The adsorption of water molecules in the external (and less ordered) part of the shell promotes a swelling effect on the polymeric network. Due to this fact the molecules of oil in these less ordered zones are displaced to the external part and, afterwards, delivered to the fabric, where the chemical affinity between the fibers and active principle will govern the release mechanism. Meanwhile, water is diffusing into the inner part of the capsule due to the internal concentration gradient created (that depends, basically, on the hydrophilicity of the fiber), which extends the swelling effect to more ordered zones and, consequently, enables the delivery of the active principle.

In the diffusion process the matter is transported to the core of the system, resulting in molecular random movements that occur across small distances. Adolf Fick, in 1855 [109], quantified the diffusion process by adopting the mathematic equation proposed by Fourier (Heat Exchange Transfer Phenomenon) [109]. The proposed Equation (1) is as follows:

(1) $$\frac{dQ}{dt}=-D\frac{dC}{dx}$$

where:

$\frac{dQ}{dt}$ = diffusion speed of the asset transported in a given time;

D= diffusion coefficient;

$\frac{dC}{dx}$ = concentration of the substance diffusing in the spatial coordinate.

### 3.2. Cyclodextrin Complexes

#### 3.2.1. Microscopy: Optical and SEM

**Figure 7:**
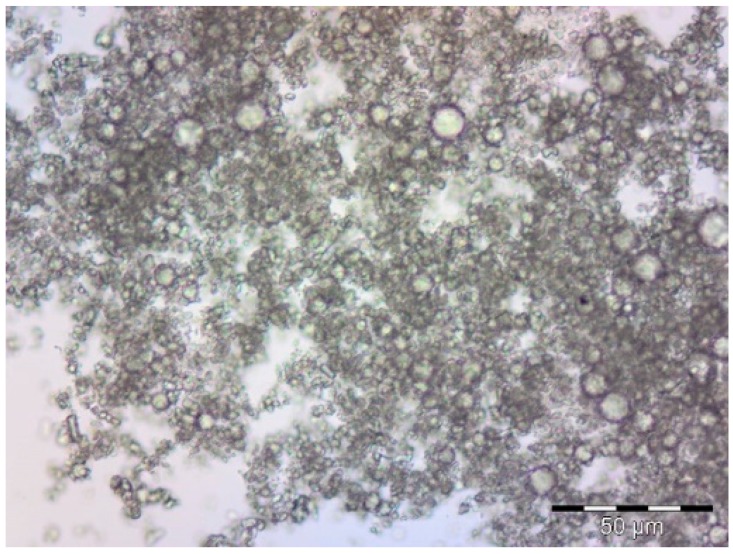
Optical microscopy image of complex. Magnification 500×.

The optical microscopy of the complexes (CD:Oil), shows their shape and distribution (Figure 7). Spherical and small cylinders are seen. The former are free citronella oil, and the latter are cyclodextrins like elongated cones. CDs can form inclusion complexes with volatile essential oils, protecting them against oxidation, enhancing their chemical stability, and reducing or eliminating eventual losses by evaporation. These results match those obtained in several other works [59,80].

**Figure 8:**
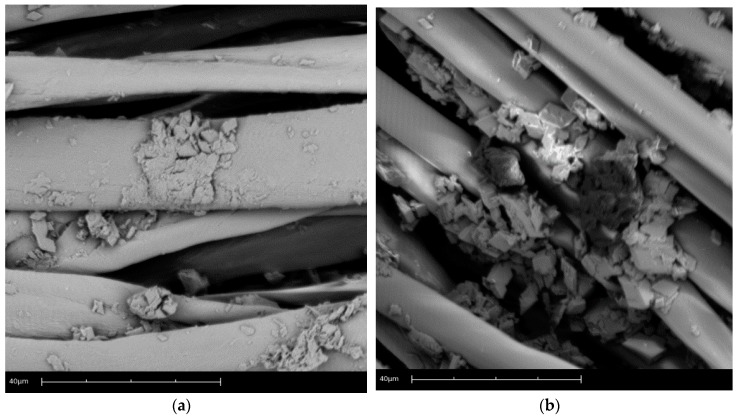
Scanning electron microscope image of complex: (**a**) cotton, and (**b**) polyester.

In Figure 8, the application of the complexes on the cotton (a) and polyester (b) surface can be seen. The CDs in textiles can create interactions according to two different mechanisms: Physical bonding without strong chemical interactions, or covalent bonding [71,82]. CDs are permanently fixed in the substrate via covalent bonds [81]. In the case of the cotton fabric, an esterification reaction promoted by the carboxyl group of the BTCA with the hydroxyl groups of the cellulose and the cyclodextrin occurs. In the polyester application, the cyclodextrin covers the fiber, forming a net over the article. In this way, cyclodextrin can adsorb bioactive molecules, making the fabric a biofunctional article.

#### 3.2.2. Drug Delivery Kinetics

**Figure 9:**
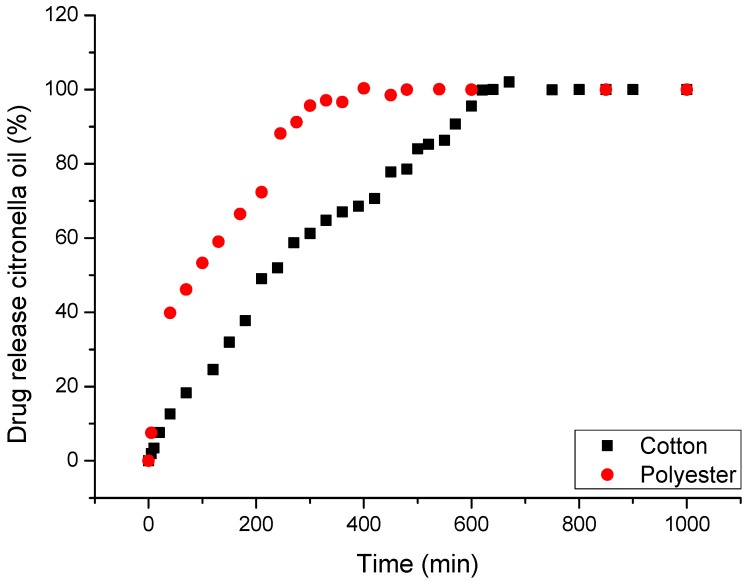
In vitro controlled release profiles in water at 37 °C for complexes (citronella: βCD) applied on textiles.

The use of textile articles as supports for controlled release requires properties to emphasize a high contact area with the skin, drug-carrier capacity, ease of application, low cost, release via stimulus, biocompatibility, non-allergenicity, and non-toxicity, among others [110,111,112,113,114]. Figure 9 shows the controlled release profile of the cotton and polyester fabrics with the complexes (CD:Oil).

The analysis shows that the release is influenced by the textile matrix and the type of polymer that constitutes it. The rate of retention of citronella oil is higher in cotton than in polyester. Although the diffusion-controlled mechanism is the same as in the case of microcapsules, here the influence of the affinity of the core substance with respect to the support substrate is high enough to modify the delivery from the complexes. The influence of the textile substrate when used in combination with vehiculizers has been studied in previous cases with microencapsulated ibuprofen [38,48,107]. It is possible to modify the diffusion of the active agent and, furthermore, to indicate that textile supports show potential for use in systems of release [115,116].

### 3.3. Liposomes

Liposomes, alone or as mixed micelles, form a very stable microstructure that allows the vehiculization of active principles for application into different textiles. The chemical and physico-chemical interactions between them and the textile substrate are translated to an adequate substantivity for most of the studied cases. However, most synthetic substrates, such as acrylic and polyester fibers, showed high desorption. This fact confirmed the preferential application of cotton and polyamide as cosmetic biofunctional textiles due to their best affinity. This study shows that polyamide always presents with high affinity for the two phospholipid structures, and also for the antioxidant [117].

The in vitro percutaneous absorption tests of the different cosmeto-textile structures that were performed (CO and PA with GA vehicularized with liposomes and mixed micelles) have been developed to show, quantitatively, the GA penetration within the layers of the skin [118].

**Figure 10:**
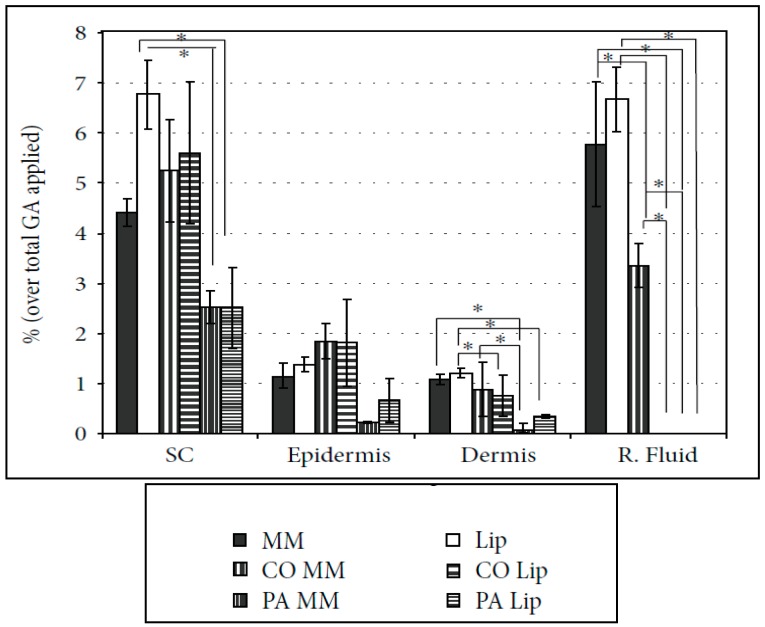
In vitro percutaneous absorption of gallic acid (GA) in liposomes (Lip) and mixed micelle (MM) formulations, and the polyamide (PA) and cotton (CO) cosmeto-textiles (SC: stratum corneum, R. Fluid: receptor fluid) (significant level accepted * *p* < 0.01).

When GA was embedded into the biofunctional textile, it always promoted a reservoir effect (concentration gradient) that was much more marked for PA. This concentration gradient is, really, responsible for managing the mass transport process of the active principle molecules to the skin. Similar penetration behavior was observed in the textiles treated with GA in MM or Lip in the different layers of skin: The stratum corneum, epidermis, and even the dermis. The GA was only detected in receptor fluid when CO was treated with the mixed micelles system (see Figure 10). 

The results show that this methodology may be useful in verifying the real amount of the encapsulated substances incorporated into textile materials that can reach the inner layers of human skin. Indeed, such materials can be considered as strategic delivery systems that can release a given active compound, controlling the final quantity delivered into the skin. If the reservoir effect is controlled, the textile substrate can act as a dosing device.

Similarly, a sun filter (EHMC) was encapsulated in different kinds of liposomic systems and applied to fabrics to create biofunctional textiles. The structured device remains stable due to the mutual affinity and the physico-chemical interactions, which can break as the fabric rubs the skin, releasing the active agents [117]. Moreover, ethyl hexyl methoxycinnamate (EHMC), was vehicularized using two liposome-based structures: Internal wool lipids (IWL) and phosphatidylcholine (PC) [118]. As in the case of GA, they were applied onto cotton and polyamide fabrics by exhaustion treatments. After topical applications on human volunteers, skin properties were evaluated by non-invasive biophysical techniques. The two methodologies, described previously, and based on percutaneous absorption, were applied to quantify the effectiveness of the penetration of the sun filter into the skin.

**Figure 11:**
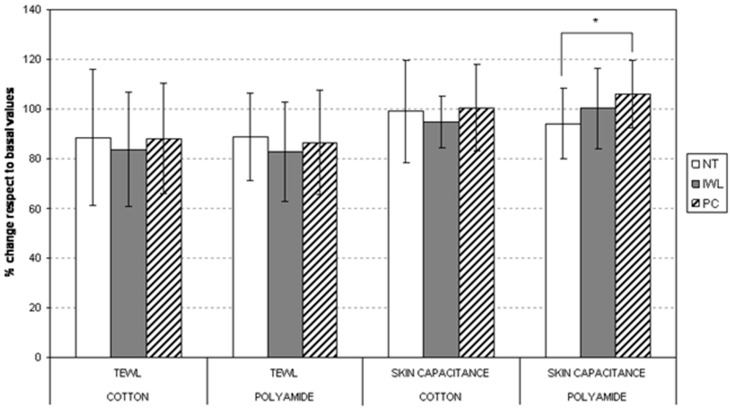
Variation of transepidermal water loss (TEWL) and skin capacitance (hydration) between initial and 24 h of skin application of cotton and polyamide fabrics. NT: non-treated fabric. Internal wool lipids (IWL): fabric treated with IWL liposomes. PC: fabric treated with PC liposomes (* *p* < 0.05 corresponds to significant difference between the marked columns).

In both lipidic systems, the absorption over the fabrics was between 10 and 15%. Nevertheless, PC-based liposomes showed a higher affinity than IWL liposomes and cotton substrates showed slightly less absorption than polyamide. The TEWL and skin capacity results are shown in Figure 11.

TEWL showed a marked decrease for IWL liposome-treated fabrics. This effect is even more marked for liposome-treated polyamide. Non-treated and PC-treated polyamide fabrics showed significant differences which will influence the mechanism of drug permeation [118].

PA-treated substrates showed good permeation into the outermost skin layers in the in vivo experiments. This is consistent with the larger effect found in the decrease in TEWL for IWL liposomes, and in the increase of hydration for PC liposomes when absorbed into polyamide fabrics. Therefore, PA substrates showed the most promising properties for a delivering device, when EHMC is vehicularized using liposomic structures. Fiber behavior in the adsorption process is very good, and enhances the permeation of EHMC through the skin. There is a greater effect of skin barrier reinforcement with IWL, and better hydration when PC liposomes are used [118].

In summary, antioxidants vehicularized through liposomes can be better applied to cotton and polyamide due to their lower desorption compared to the other fibers assayed, such as acrylic or polyester. The two in vitro and in vivo methodologies used to determine the content of active principle penetration into the skin when in contact with the smart textiles indicates the influence of the physicochemical properties of the drugs. When GA is vehicularized in liposomes into the textile, there is a clear reservoir effect that is much more marked with PA [117]. However, when a clear lipophilic compound such as EHMC is also vehicularized in liposomes in the textile, a significantly greater release of the active to the skin was found. Polyamide fibers show the higher desorption capability [118]. This was corroborated by the in vivo results of percutaneous penetration, and the greater skin barrier reinforcement and hydration of the polyamide smart textiles. Therefore, it can be concluded that there are notably different release behaviors of hydrophilic drugs, which may be retained in the hydrophilic core of the liposome in front of lipophilic drugs which are embedded in the surface lipidic bilayer of the liposomes, favoring their release [119].

## 4. Conclusions

As can be seen from the experimental results obtained, many possibilities exist to make “active” textiles substrates in different environments, using well-designed vehicularizing systems to incorporate these into the fabrics. These complex structures can be, among others, microcapsules, cyclodextrins, or liposomes.

The textile substrate played a very important role in every system tested. The chemical affinity shown in every case clearly controlled the whole release behavior. The in vitro tests are useful to define every step of the delivery mechanisms.

The same citronella oil, vehicularized with different chemical structures, and applied to the same textile substrates, presented different release behaviors. This opens many possibilities for the design of biofunctional textiles.

The use of thermogravimetric methods allows us to define and establish the level of interactions between the active principle and the shell material. 

In general, the use of vehicles and textile substrates improves the absorption of complex molecules and formulations by the skin. 

The response of the smart fabric depends on the existing interactions between the active principle and the molecular covered structure, and on the interactions between these components and the textile substrate. The combination of these effects results in the development of biofunctional textiles capable of combining specific characteristics of bioactive molecules that cannot be inserted directly into the fabric, as is the case in essential oils that lose their effect due to their volatility.

Therefore, the use of biofunctional textiles allows the treatment of many skin diseases via skin–textile contact, displaying advantages in relation to the administration of the active substance.

## Figures and Tables

**Table 1:** Polymers used in microencapsulation [21,22,23,24,25,26,27].

Source	Polymer
Natural Polysaccharides	Starch, cellulose, chitosan, gum arabic, and alginate
Modified Polysaccharides	Dextrins, carboxymethylcellulose, ethylcellulose, methylcellulose, acetylcellulose, and nitrocellulose
Proteins	Gluten, casein, gelatin, and albumin
Waxes and lipids	Paraffin, tristearine, stearic acid, monoacyl, and diacyl
